# Intestinal endothelial cells increase HIV infection and latency in resting and activated CD4 + T cells, particularly affecting CCR6 + CD4 + T cells

**DOI:** 10.1186/s12977-023-00621-y

**Published:** 2023-05-18

**Authors:** Jessica Eddy, Fisher Pham, Rachel Chee, Esther Park, Nathan Dapprich, Stacy L. DeRuiter, Anding Shen

**Affiliations:** 1 Department of Biology, Calvin University, 3201 Burton St. SE, Grand Rapids, MI 49546 USA; 2 Department of Mathematics & Statistics, Calvin University, 3201 Burton St. SE, Grand Rapids, MI 49546 USA

**Keywords:** HIV, Resting CD4 + T, Latent reservoir, Endothelial cells, Viral reservoir, CCR6 + Th17 cell, Gut associated lymphoid tissue

## Abstract

**Background:**

With suppressive antiretroviral therapy, HIV infection is well-managed in most patients. However, eradication and cure are still beyond reach due to latent viral reservoirs in CD4 + T cells, particularly in lymphoid tissue environments including the gut associated lymphatic tissues. In HIV patients, there is extensive depletion of T helper cells, particularly T helper 17 cells from the intestinal mucosal area, and the gut is one of the largest viral reservoir sites. Endothelial cells line lymphatic and blood vessels and were found to promote HIV infection and latency in previous studies. In this study, we examined endothelial cells specific to the gut mucosal area—intestinal endothelial cells—for their impact on HIV infection and latency in T helper cells.

**Results:**

We found that intestinal endothelial cells dramatically increased productive and latent HIV infection in resting CD4 + T helper cells. In activated CD4 + T cells, endothelial cells enabled the formation of latent infection in addition to the increase of productive infection. Endothelial-cell-mediated HIV infection was more prominent in memory T cells than naïve T cells, and it involved the cytokine IL-6 but did not involve the co-stimulatory molecule CD2. The CCR6 + T helper 17 subpopulation was particularly susceptible to such endothelial-cell-promoted infection.

**Conclusion:**

Endothelial cells, which are widely present in lymphoid tissues including the intestinal mucosal area and interact regularly with T cells physiologically, significantly increase HIV infection and latent reservoir formation in CD4 + T cells, particularly in CCR6 + T helper 17 cells. Our study highlighted the importance of endothelial cells and the lymphoid tissue environment in HIV pathology and persistence.

## Background

With suppressive antiretroviral therapy, HIV infection is well-managed in most patients. However, eradication and cure are still beyond reach, and lifelong therapy is required, mainly due to the latent viral reservoir in CD4 + T helper cells. With a long lifespan and an extremely slow decay rate [1, 2], this reservoir ensures viral persistence in infected patients. The vast majority of HIV latent reservoirs are located within lymphoid tissues, and the gastrointestinal tract is one of the largest tissue reservoirs [3].

Intestinal pathology is prominent in HIV patients. In many HIV patients, even with suppressive therapy, persistent inflammation and gut pathology still cause significant morbidities [4]. The gut associated lymphoid tissue (GALT) is considered the largest immunological site in the body and harbors the largest number of T cells [5]. Up to 90% of lymphocytes reside in the GALT [6]. In the early stages of HIV and SIV (simian immunodeficiency virus) infection, viral replication in the intestinal region is extremely high, and most of the CD4 + T cell population in the gut is depleted due to toxicity from infection or immune responses [7–10]. High-level viral replication in the GALT and loss of CD4 + T cells result in the disruption of the mucosal barrier and bacterial translocation [7, 11], which then results in persistent systemic inflammation. Upon antiretroviral therapy, CD4 + T cell populations are restored in blood and in lymph nodes, but in most patients, they are either delayed or not restored in the gut mucosal area, leading to continued microbial translocation and inflammation [10, 12]. Even with successful anti-retroviral therapy, HIV still persists in the GALT [13, 14], and the intestinal lymphoid tissue harbors a significant latent viral reservoir [15, 16]. From clinical observations and animal studies, viral pathology and reservoir formation in the gastrointestinal tract are well-documented, even with successful anti-retroviral therapy, but the mechanisms are still poorly understood.

Studying HIV pathogenesis and latent reservoir formation in the lymphoid tissue such as the GALT directly is difficult. Most of what we understand about HIV pathogenesis and latent reservoir formation in T cells has come from in vitro studies and from peripheral blood cells, but CD4 + T cells within the lymphoid tissues can behave very differently due to the influences from surrounding cells and soluble factors. For example, mainly based on in vitro experiments, with regard to the latent reservoir formation in resting CD4 + T cells, it is understood that HIV cannot complete integration in resting CD4 + T cells; rather, activated CD4 + T cells are infected and revert to a resting phenotype with integrated provirus [17–19]. However, studies done in vivo or ex vivo showed that resting CD4 + T cells can be infected in the context of lymphoid tissues [7, 20–26]. One of the studies found that resting CD4 + T cells support HIV replication in lymphoid tissue (tonsil) explants, whereas purified tonsillar resting CD4 + T cells did not support HIV replication [25]. Another study found that when infected ex vivo, CD4 + T cells isolated from splenic and tonsillar lymphoid tissues had significantly higher latent infection rates when compared to purified CD4 + T cells isolated from peripheral blood [27]. We and others have demonstrated that while resting CD4 + T cells were poorly infected by themselves, after being stimulated by endothelial cells (EC), they can be directly infected while remaining in a resting state, resulting in substantially higher productive and latent infections [28–33]. EC are non-hematopoietic stromal cells that line the blood and lymphatic vessels in secondary lymphoid tissues and have constant interactions with CD4 + T cells trafficking through them. We have set up an EC and CD4 + T cell co-culture model to study the effect of lymphoid tissue, particularly endothelial cells, on T cells within the context of HIV infection and latency formation.

EC are a diverse population of cells, including both macrovascular EC and microvascular EC. Macrovascular EC, such as human umbilical cord vascular endothelial cells (HUVEC), are involved in the formation of large vessels. Microvascular EC, such as lymphatic endothelial cells (LEC), mainly line the wall of blood and lymphatic vessels in lymphoid tissues. Each tissue has its own corresponding EC type. We have demonstrated the effect of both macrovascular EC (HUVEC, [28, 29]) and microvascular EC (LEC, [30]) on HIV infection of resting CD4 + T cells. In this study, we set up a model to investigate the EC type specific for the gut: Human Intestinal Microvascular Endothelial Cells.

EC are abundant in gut mucosal tissues and are in frequent contact with T cells that are destined for the intestines. EC in vivo express MHC class II and co-stimulatory molecules such as CD58 and are considered antigen presenting cells. We hypothesized that intestinal endothelial cells (IEC) are likely involved in stimulating resting T cells in the gut, facilitating HIV infection and latency formation, and contributing to cell depletion and pathogenesis in the GALT, as found in clinical observations.

Due to their proximity to antigens and frequent encounters with antigens, CD4 + T cells in the gut are often in an activated state [34]. Activated CD4 + T cells are much more susceptible to HIV infection than resting CD4 + T cells. However, the presence of latent infection in activated CD4 + T cells remains unclear. Some found there to be a latent reservoir in the activated CD4 + T cells although resting cells displayed a higher propensity for latent infection [27], while others found there to be no latent infection in activated CD4 + T cells [35]. Some found infecting activated CD4 + T cells after their peak of activation generated higher levels of latent infection [19]. Our own data showed the lack of latent infection in activated CD4 + T cells when they were infected at the peak of their activation [28]. Therefore, we also investigated the effect of IEC on HIV infection and latency formation in activated CD4 + T cells in addition to resting T cells.

CD4 + T cells in the mucosal area exhibit a memory phenotype [34] and have increased expression of CCR5, a co-receptor for HIV. HIV uses CD4 as a receptor and a chemokine receptor such as CXCR4 (X4) or CCR5 (R5) as a co-receptor for attachment on a target cell. Based on co-receptor usage, HIV-1 can be subtyped as X4 tropic virus or R5 tropic virus. R5 tropic viruses dominate early infection in an individual and are frequently the ones to be transmitted, and X4 tropic viruses arise later in the infection course [36]. We have been using an X4-tropic GFP reporter virus in prior studies since resting CD4 + T cells express CXCR4 constitutively but very little CCR5. In this study, for activated and memory CD4 + T cells, we also used an R5 tropic reporter virus for infection, as this subtype has more in vivo relevance in the mucosal area.

Lastly, Th17 cells are a subpopulation of CD4 + T cells that are abundant in the intestinal mucosal tissue [37]. Th17 cells are characterized by the secretion of IL-17 and by the surface chemokine receptor CCR6 [38], which binds to chemokine CCL20 and aids in migration to the intestinal tissues [39]. Upon HIV infection, Th17 cells experience preferential and nearly complete depletion from the gut [40, 41]. Their functions within the gut mucosa are altered and, even after anti-retroviral therapy, their numbers or functions are not restored [42]. Th17 cells have been shown to feature higher HIV infection rates than other CD4 + T cells [43], due to a variety of surface and intracellular factors that aid in the entry and replication of HIV (reviewed in [44]). We investigated the effect of IEC on HIV infection of CCR6 + CD4 + T cells in this study.

## Results

### IEC induce productive HIV infection in resting CD4 + T cells

**Figure 1:**
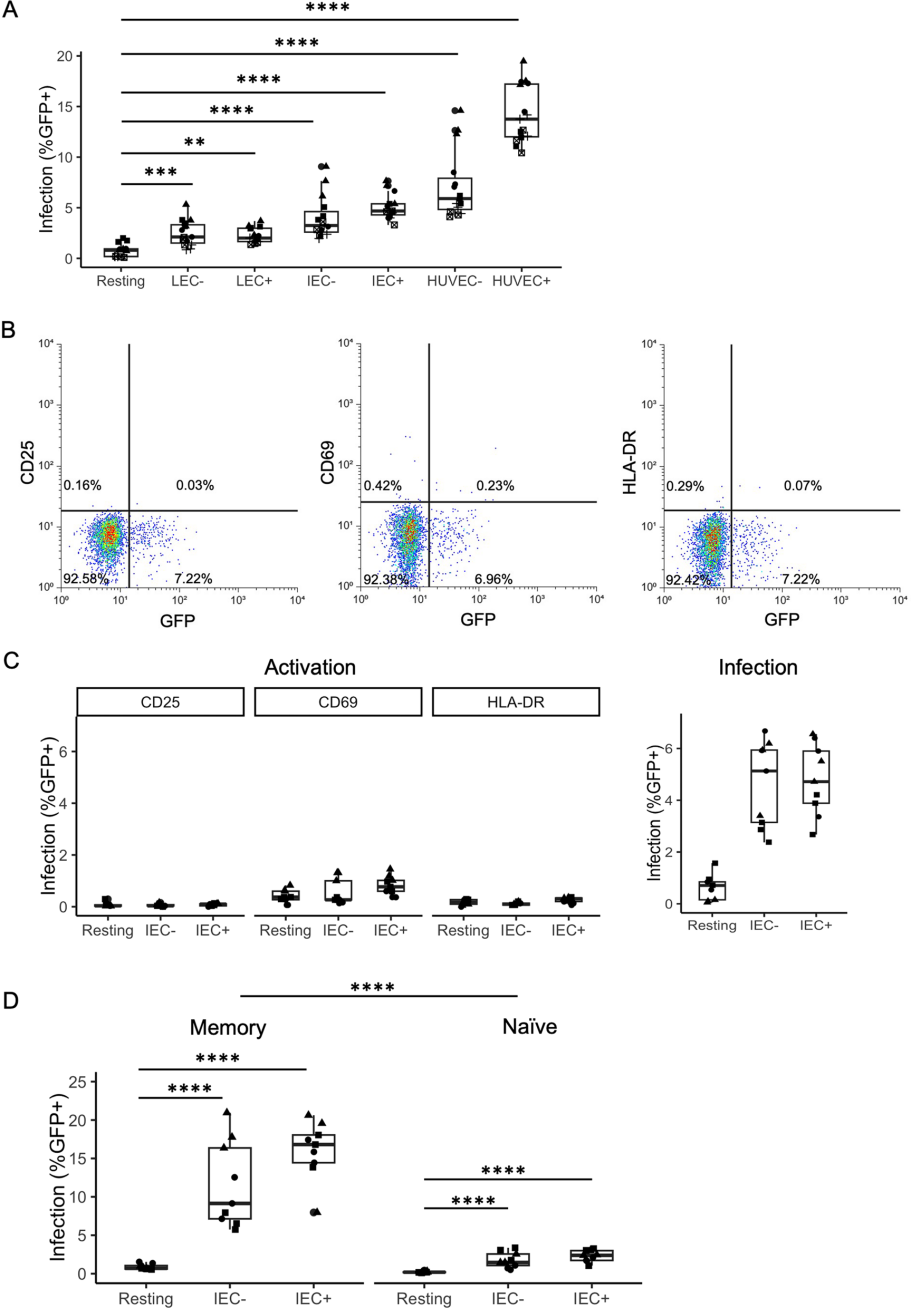
**Fig. 1** IEC stimulation increases HIV infection of resting CD4 + T cells. Here and throughout the paper, boxplots show the median (horizontal line), and the central box extends to the 25th and 75th percentiles. The whiskers then extend 1.5 inter-quartile ranges or to the minimum or maximum observed value, whichever is closer to the median. **A** Infection rates in T cells stimulated with endothelial cells. Resting T cells were cultured alone (Resting), or co-cultured with human umbilical vein endothelial cells (EC), human lymphatic endothelial cells (LEC), or human intestinal endothelial cells (IEC). + and − indicate treatment with or without IFN-γ respectively in EC, LEC, or IEC. All T cells were infected with an HIV reporter virus expressing GFP 1 day after co-culture, and the %GFP+ cells were measured on day 6 post-infection. Samples were taken in triplicate for each donor, and different donors are represented by different symbols (n = 5). P-values are from post-hoc pairwise comparisons of marginal means based on beta generalized linear models (*p<0.05, **p<0.01, ***p<0.001, ****p<0.0001). **B** Expression of activation markers versus GFP expression in IEC + stimulated resting CD4 + T cells. Similar to A, but on day 6 post-infection, T cells were stained for three activation markers: CD25, CD69, and HLA-DR. **C** Expression of activation markers and infection rates in T cells with and without IEC stimulation (n = 3), similar to (**B**). **D** Infection rates in resting memory and naïve T cells stimulated with IEC. Memory and naïve T cells were isolated, and the rest of the experimental procedures are the same as (**A**). Samples were taken in triplicate for each donor, and different donors are represented by different symbols (n = 3). P-values are from post-hoc pairwise comparisons of marginal means based on beta generalized linear models (****p<0.0001).

The gut is a major site for HIV infection and pathogenesis. A large number of CD4 + T cells are infected in the gut mucosal area resulting in CD4 + T cell depletion. Resting CD4 + T cells generally resist HIV infection, but EC simulation was found to dramatically increase HIV infection of those cells [28, 30–32]. IEC reside abundantly in the gut associated lymphatic tissues (GALT) and are in frequent contact with CD4 + T cells. We investigated the effect of IEC in inducing productive HIV infection in resting CD4 + T cells. Resting CD4 + T cells were isolated from HIV-negative donors and co-cultured with various types of EC: IEC + and IEC −, LEC + and LEC −, and HUVEC (HUVEC + and HUVEC −). “+” and “−” indicate EC pre-treated with or without IFN- γ respectively. EC isolated ex vivo lose the expression of MHC II, and treatment of IFN- γ for 3 days restores the expression of MHC II. After EC were plated for at least 3 h, EC media were removed before CD4 + T cells were added to the co-culture with T cell media (RPMI + FBS). After one day of co-culturing, CD4 + T cells were infected with a GFP reporter virus, and infection rates (% GFP+) were examined on day 6 post-infection. We found IEC (both + and −) induced significantly higher levels of infection than resting CD4 + T cells alone (Fig. 1A, p < 0.0001). We also compared the infection rates in IEC-stimulated T cells with those stimulated by Human Umbilical Cord Vascular Endothelial Cells (HUVEC) and Lymphatic endothelial cells (LEC), the two EC types associated with inducing HIV infection in resting CD4 + T cells shown previously. All three types of EC increased HIV productive infection in resting T cells, although the levels of increase showed some variation reflecting the heterogeneity among different types of EC.

### IEC induce productive infection in resting CD4 + T cells without causing activation

Activated CD4 + T cells are much more susceptible to HIV infection than resting CD4 + T cells. To examine whether IEC stimulation would activate resting CD4 + T cells, we measured cell activation markers CD25, CD69, and HLA-DR in IEC-stimulated resting CD4 + T cells on day 6 post-infection using fluorescently labeled antibodies against these markers and flow cytometry. As shown in (Fig. 1C), less than 1% of the CD4 + T cells co-cultured with IEC − or IEC + expressed any activation marker. In CD4 + T cells co-cultured with IEC +, there were typically slightly more cells expressing activation markers than those cultured alone or with IEC −. Even in IEC + stimulated T cells, the majority of infected (GFP +) T cells do not express any activation markers (Fig. 1B). These CD4 + T cells may recognize allogeneic MHC II on IEC + and become activated. A similar phenomenon was observed with HUVEC and LEC stimulation. However, the proportion of CD4 + T cells that were infected was always significantly higher than the proportion of cells that were activated (comparing levels of activation versus levels of infection in Fig. 1C). These results show that T cell activation is not a mechanism for increased HIV infection in IEC-stimulated resting T cells.

### IEC induce more infection in memory CD4 + T cells than in naïve CD4 + T cells

In vivo, memory CD4 + T cells are preferentially infected and harbor most of the latent reservoir. Therefore, we examined whether IEC stimulation has more effect on the memory population or the naïve population of resting CD4 + T cells. We isolated CD4 + CD45RO- naïve resting T cells and CD4 + CD45RA- memory resting T cells from the same donor, and co-cultured them with IEC one day before infection. Infection rates (%GFP+) were examined on day 6 post-infection. We found that memory CD4 + T cells were infected at much higher rates than naïve CD4 + T cells in general (Fig. 1D, overall comparison between memory and naïve T cells, p < 0.0001). Although both memory and naïve CD4 + T cells co-cultured with IEC still showed greater infectivity than those CD4 + T cells cultured alone (Fig. 1D, p < 0.0001), the increases in infection for memory T cells were much higher than for naïve T cells: IEC − cocultures increased infection by > 10% on average in memory T cells compared with 1.5% in naïve T cells; likewise, IEC + cocultures increased infection by > 15% on average in memory T cells compared with 2% in naïve T cells. This suggested that signals provided by IEC to memory CD4 + T cells were able to overcome the restrictions to a much greater extent than in naïve cells. This is consistent with the fact that endothelial cells express CD58 but not the co-stimulatory molecules CD80/86 and thus are better at stimulating memory CD4 + T cells than naïve CD4 + T cells. Naïve CD4 + T cells generally require a stronger co-stimulatory signal (e.g. through CD80/86) for activation than memory CD4 + T cells.

### IL-6 is involved in IEC promotion of productive infection in resting CD4 + T cells

**Figure 2:**
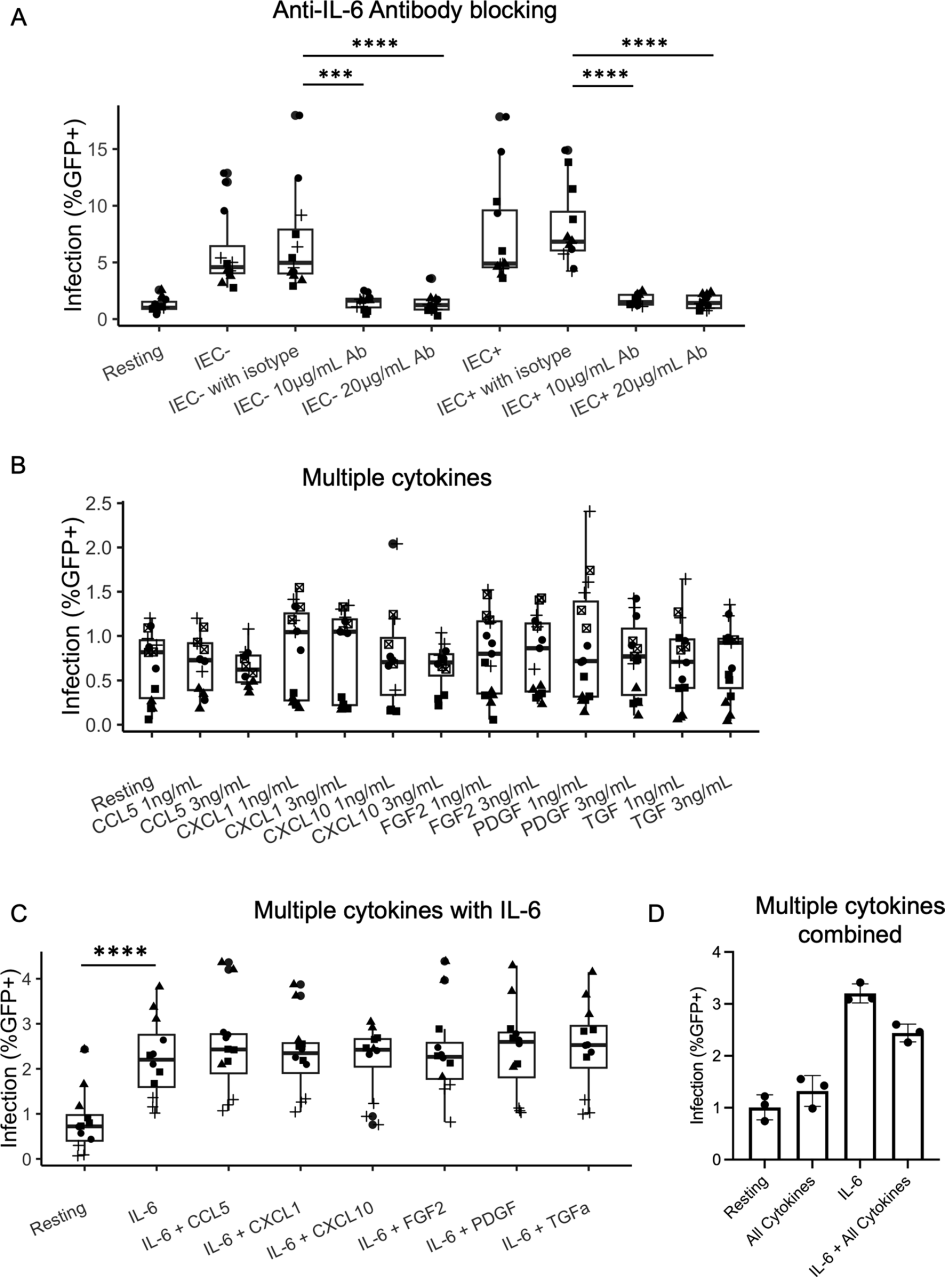
**Fig. 2** Involvement of IL-6 and other cytokines in IEC stimulation of resting CD4 + T cells.**A** Blocking IL-6 with antibody in IEC co-culture with CD4 + T cells. Resting CD4 + T cells were cultured alone or with IEC +/− . At the time of co-culture, anti-human IL-6 antibody was added at 10 and 20µg/mL. Isotype control antibody at 20µg/mL was also included in separate wells as negative controls. All T cells were infected with an HIV reporter virus expressing GFP one day after co-culture. %GFP + cells were measured on day 6 post-infection. **B** Infection rates in resting CD4 + T cells treated with various cytokines. Resting CD4 + T cells were isolated from healthy blood donors and treated with various cytokines (FGF-2, CXCL10, CXCL1, PDGF-BB, TGF-alpha, and CCL5) at concentrations as indicated for one day before infection with an HIV reporter virus expressing GFP. Infection rates were measured 6 days after infection. **C** Infection rates in resting CD4 + T cells treated with various cytokines in combination with IL-6. Similar to (**B)**, except treated with recombinant IL-6 (10ng/mL) and one of the six cytokines (FGF-2, CXCL10, CXCL1, PDGF-BB, TGF-alpha, and CCL5, 3ng/mL). Samples were taken in triplicate for each donor, and different donors are represented by different symbols (**A** n = 4, **B** n = 5, **C** n = 4). P-values are from post-hoc pairwise comparisons of marginal means based on beta generalized linear models (***p<0.001, ****p<0.0001). **D** Infection rates in resting CD4 + T cells treated with all cytokines and in combination with IL-6. Similar to C, except treated with IL-6 (10ng/mL), all five cytokines (FGF-2, CXCL10, CXCL1, PDGF-BB, TGF-alpha, and CCL5, 3ng/mL), or the combination of IL-6 and all five cytokines. Samples were taken in triplicates and means+/− standard errors are plotted. Data shown are representative of two independent experiments.

The pro-inflammatory cytokine IL-6 was found to be produced by HUVEC and LEC and was involved in HUVEC/LEC stimulation of resting CD4 + T cells [29, 30]. To examine the role of IL-6 in IEC stimulation, we introduced an anti-IL-6 antibody in IEC-T cell co-cultures. IEC were plated for at least 3 h before resting CD4 + T cells were added to the IEC after supernatants were removed from the plated IEC. At the same time, an anti-human IL-6 antibody was added to the co-culture at various concentrations (10 and 20 µg/mL). Isotype control antibodies were also included in separate wells as a negative control. After one day, CD4 + T cells cultured alone, co-cultured with IEC with or without anti-IL-6 antibody, were infected. Infection (%GFP+) levels were measured on day 6 post-infection. As seen in Fig. 2A, the addition of anti-IL-6 antibody resulted in significantly lower infection rates in resting cells stimulated by both IEC − and IEC + (p < 0.001 for both comparisons). In most cases, the addition of anti-IL-6 antibody reduced the infection rates to the level of unstimulated resting CD4 + T cells, suggesting that IL-6 was necessary for the effect of IEC stimulation.

### Lack of effect with other cytokines in the stimulation of resting CD4 + T cells with or without the addition of IL-6

Even though IL-6 seemed to be necessary for the effect of IEC stimulation, it did not appear to be sufficient by itself. Treatment of resting CD4 + T cells with recombinant IL-6 did not induce the same level of HIV infection as in EC stimulation [29]. A multiplex cytokine analysis was performed in search of additional soluble factors potentially involved in EC stimulation of resting CD4 + T cells. Supernatants from resting CD4 + T cells cultured alone, co-cultured with HUVEC, or co-cultured with LEC were collected and analyzed with a multiplex cytokine panel consisting of 65 cytokines and chemokines (Eve Technologies Corporation). FGF-2, CXCL10, CXCL1, PDGF-BB, TGF-alpha, and CCL5 were among the cytokines found to be up-regulated in LEC/HUVEC co-cultures compared with resting CD4 + T cells alone. To examine whether these cytokines were involved in IEC stimulation of resting T cells, we treated resting CD4 + T cells with recombinant cytokines FGF-2, CXCL1, CXCL10, PDGF-BB, TGF-α and CCL5 at various concentrations (1ng/mL and 3ng/mL), all well above their levels in co-culture supernatants. Compared with resting CD4 + T cells alone, none of the cytokine treatments induced additional infection (Fig. 2B). We also treated resting CD4 + T cells with FGF-2, CXCL10, CXCL1, PDGF-BB, TGF-alpha, and CCL5 in combination with IL-6 (10ng/mL). While IL-6 induced higher infection rates than resting CD4 + T cells alone, the addition of other cytokines did not induce additional infection (Fig. 2C). Finally, we treated resting CD4 + T cells with the combination of all five cytokines and in some wells with IL-6 as well. Still, we did not observe any increased infection by those cytokines, aside from IL-6 (Fig. 2D).

### IEC promotion of productive infection in resting CD4 + T cells does not involve CD2

**Figure 3:**
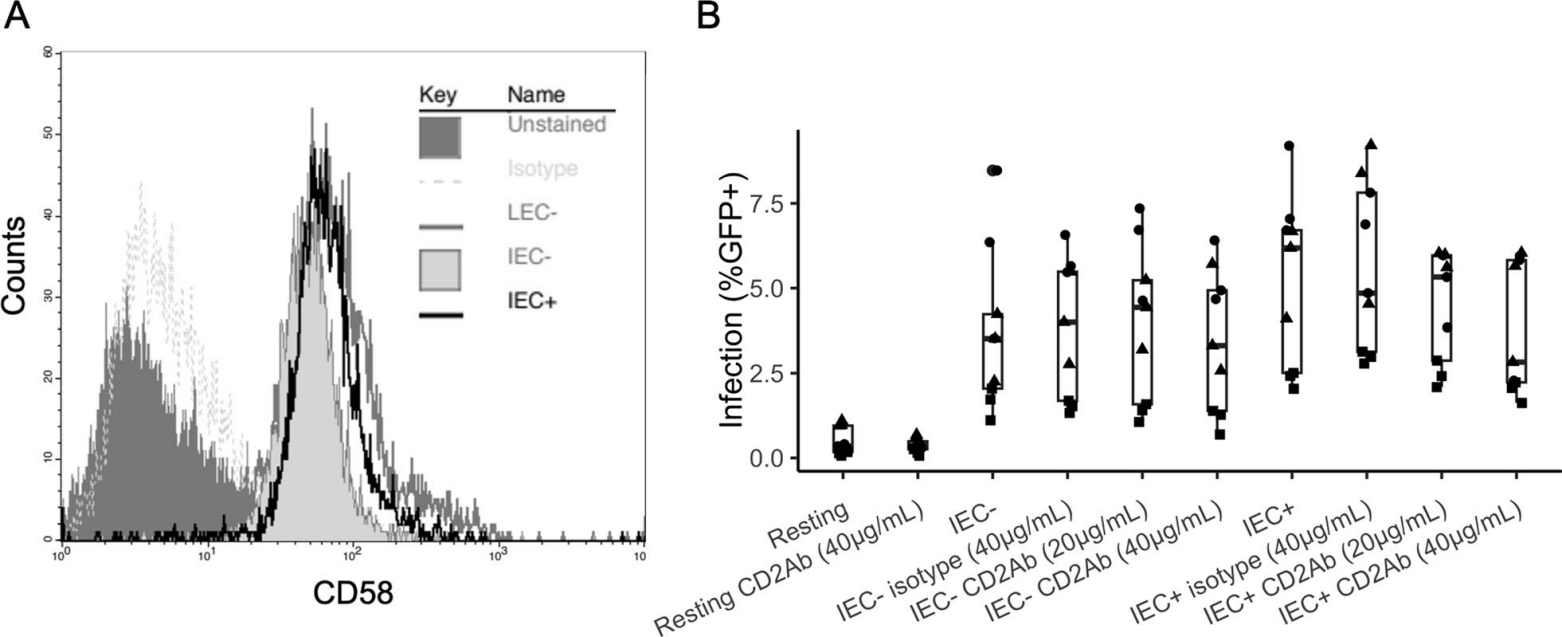
**Fig. 3** IEC induction of productive infection does not involve CD2. **A** Expression of CD58 in IEC +/− and LEC −. IEC +, IEC − and LEC − were stained with anti-CD58 antibodies. Isotype control was included as a negative control. **B** CD2 blocking experiment in IEC-stimulated resting CD4 + T cells. Resting CD4 + T cells were cultured alone or co-cultured with IEC +/−. One hour before co-culturing with IEC, CD2 blocking antibody was added at various concentrations (20 and 40µg/mL) to resting CD4 + T cells. Isotype control antibody (at 40µg/mL) was also included as a negative control. All CD4 + T cells were infected with an HIV reporter virus expressing GFP one day after co-culture. Infection rates (%GFP + cells) were measured on day 6 post-infection. Samples were taken in triplicate for each donor, and different donors are represented by different symbols (n = 3). P-values from post-hoc pairwise comparisons of marginal means based on beta generalized linear models were not significant between isotype controls and CD2 treatments.

CD2-CD58 interaction has been found to be involved in EC stimulation of resting CD4 + T cells [28, 31, 32]. CD58 is a known co-stimulatory molecule that binds to CD2 on CD4 + T cells. However, although LEC express CD58, CD2 blocking experiments showed that CD58-CD2 interaction was not involved in LEC stimulation of resting CD4 + T cells [30]. IEC + and IEC − express CD58 at a level similar to LEC (Fig. 3A). To examine whether CD2 was involved in IEC stimulation, we use an anti-CD2 antibody in IEC-T cell co-cultures. Resting CD4 + T cells were incubated with anti-CD2 antibody (at 20 µg/mL and 40 µg/mL) for at least 0.5 h before being added to plated IEC. Isotype control antibodies were also included in separate wells as a negative control. After one day, CD4 + T cells cultured alone, stimulated by IEC, with or without anti-CD2 antibody, were infected, and GFP levels were measured on day 6 post-infection. In IEC- stimulated CD4 + T cells, blocking CD2 had no effect on infection rates. For IEC + stimulated CD4 + T cells, there were consistently no effects or just a slight decrease (not statistically significant) in infection rates with CD2 antibodies compared with isotype controls (Fig. 3B).

### IEC increase latent viral infection in resting CD4 + T cells

**Figure 4:**
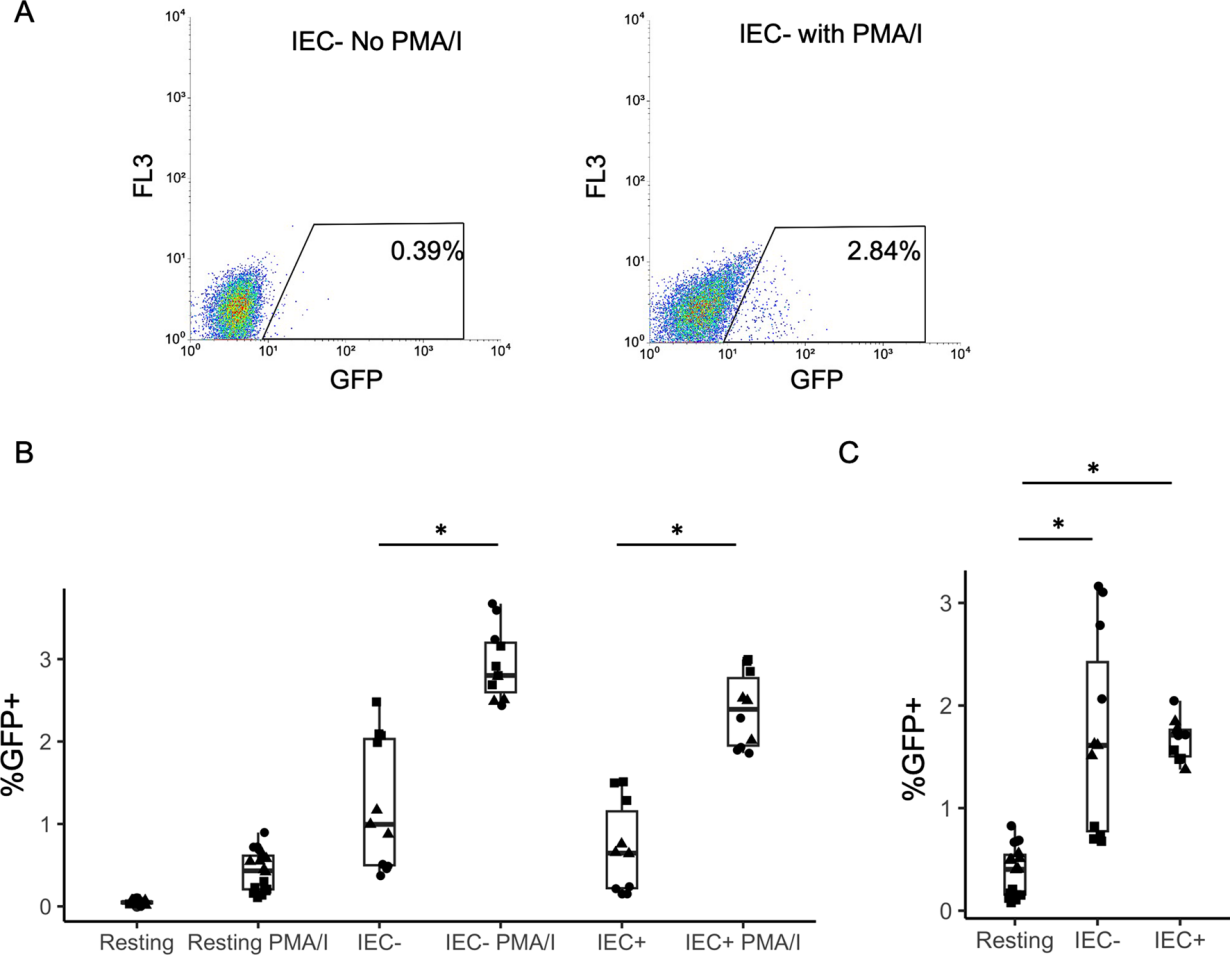
**Fig. 4** Resting CD4 + T cells were cultured alone or with human intestinal endothelial cells (IEC). + and − indicate treatment with or without IFN-γ respectively in IEC. All CD4 + T cells were infected with an HIV reporter virus expressing GFP 1 day after co-culture. On day 8 post-infection, GFP negative cells were sorted and cultured with or without PMA/Ionomycin for 16hr. %GFP + cells were measured 2 days after PMA/I activation. Raltegravir (at 3.3μM) was included in the culture media for both PMA/I treated and untreated CD4 + T cells.**A** GFP expression levels in PMA/I stimulated and unstimulated resting T cells co-cultured with IEC −. **B** GFP expression levels in resting CD4 + T cell alone, IEC + and IEC − co-cultures with and without PMA/I stimulation. Samples were taken in quadruplicate or triplicate for each donor, and different donors are represented by different symbols (n = 3). **C** Latent infection in resting CD4 + T cell alone, IEC + and IEC − co-cultures. Same experiment as B. Latent infections were calculated by the difference in GFP levels between PMA/I treated and untreated samples. P-values are from post-hoc pairwise comparisons of marginal means based on beta generalized linear models (*p<0.05).

So far, we have seen that IEC promoted productive infection in resting CD4 + T cells. We wanted to know whether IEC stimulation would result in latent infection of resting CD4 + T cells, as the gut mucosal tissue was found to be a major site for HIV latent reservoir [15, 16]. Latently infected cells have integrated intact virus but do not have viral gene expression. In our model, we use GFP expression to represent viral gene expression. Latent infection can be reactivated via CD4 + T cell activation such as through PMA/Ionomycin stimulation. To investigate latent infection, we cultured resting CD4 + T cells with IEC for one day and infected them with the GFP reporter virus. On day 8 post-infection, when most unintegrated viral DNA had decayed and a majority of the integrated virus had expressed GFP, productively infected (GFP +) cells were removed and GFP negative cells were collected via flow cytometric sorting. The GFP negative cells were cultured alone or with phorbol myristate acetate (PMA, 10ng/mL) plus Ionomycin (I, 1 µg/mL) for 16 h. Sixteen hours later the PMA/I was removed and replaced with regular media, and the following day GFP expressions were examined for CD4 + T cells treated with or without PMA/I using flow cytometry. PMA/I stimulation is known to activate T cells and reactivates latent virus to express GFP. The difference in GFP expression between PMA/I treated and untreated cells represents latent infection. To prevent de novo integration and to ensure post-integration latency was detected, an integrase inhibitor Raltegravir (at 3.3µM) was included in the culture media for both PMA/I treated and untreated CD4 + T cells. As shown in Fig. 4A, there was very little GFP expression in cells without PMA/I stimulation, but there was a substantial increase of GFP expression after PMA/I stimulation, demonstrating the expression of latent virus upon activation of CD4 + T cells. There was a small (did not achieve statistical significance p = 0.2) increase of GFP expression after PMA/I stimulation for resting CD4 + T cells alone, but a significant increase was observed in both IEC − and IEC + stimulated resting CD4 + T cells (Fig. 4B, IEC − p = 0.034 and IEC + p = 0.016). The difference of GFP expressions in PMA/I stimulated CD4 + T cells versus unstimulated CD4 + T cells represented latent infection. The differences were calculated and compared between resting CD4 + T cells cultured alone and co-cultured with IEC. The increase of latent infection in IEC stimulated CD4 + T cells compared with resting CD4 + T cells alone was statistically significant for both IEC − and IEC + (Fig. 4C, p values 0.025 and 0.028 respectively), showing that IEC stimulation results in significantly more latent infection in resting CD4 + T cells.

### IEC increase productive infection in activated CD4 + T cells

**Figure 5:**
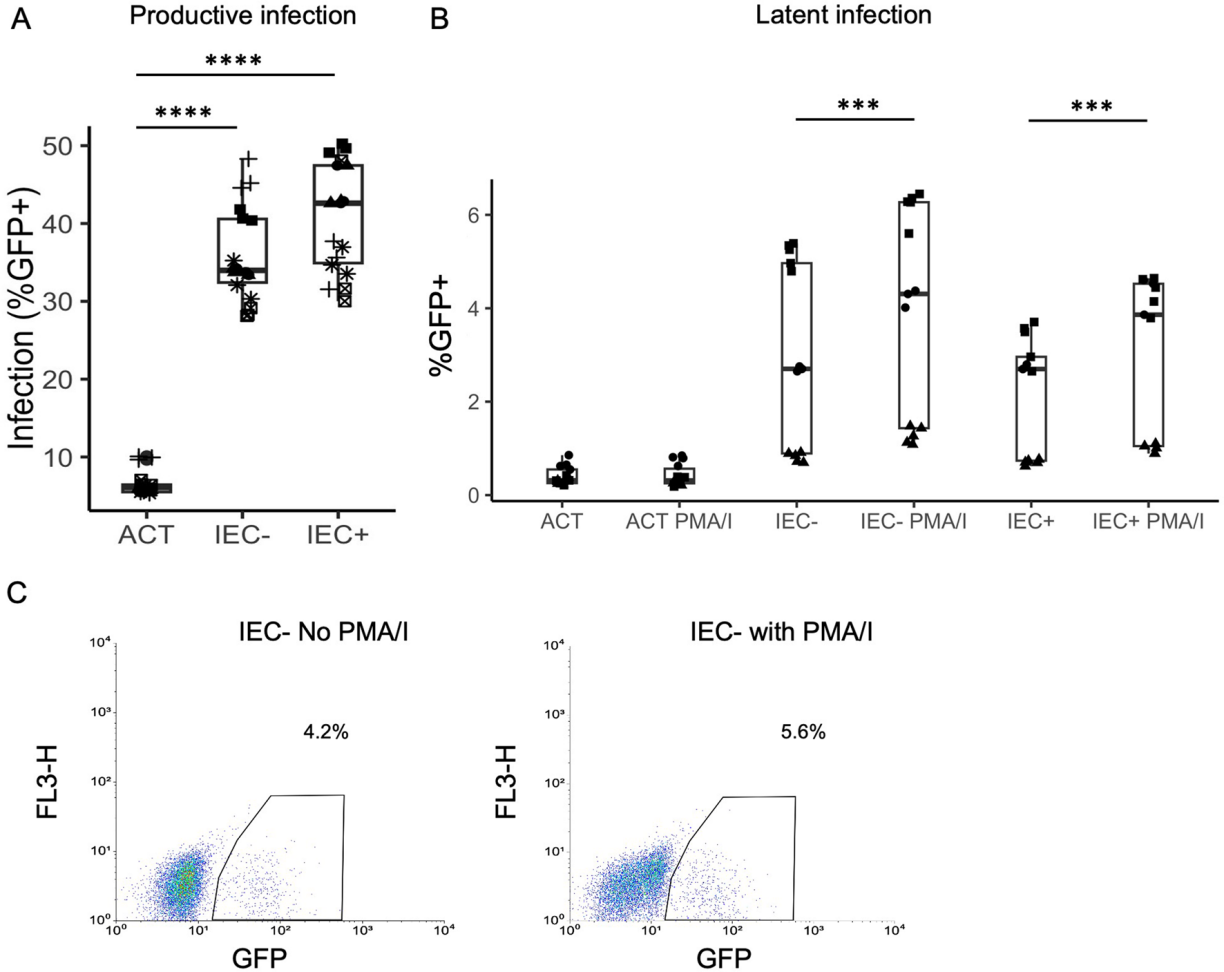
**Fig. 5** IEC stimulation increases infection rates in activated CD4 + T cells. PBMC were activated with PHA (1mg/mL) and IL-2 (10ng/mL) for 2 days before isolating CD4 + T cells by bead depletion. On day 6 post-activation, the activated CD4 + T cells were either cultured alone, or co-cultured with IEC −, or IEC +. All T cells were infected with an HIV reporter virus expressing GFP one day after co-culture. **A** Productive infection. %GFP + cells were measured 3 days post-infection. Samples were taken in triplicate for each donor, and different donors are represented by different symbols (n = 6). P-values are from post-hoc pairwise comparisons of marginal means based on beta generalized linear models (****p<0.0001). **B** Latent infection. Five days after CD4 + T cells were infected, GFP − cells were sorted and cultured with or without PMA/I stimulation. GFP expression was measured 2 days later. Raltegravir (at 3.3μM) was included in the culture media for both PMA/I treated and untreated CD4 + T cells. Samples were taken in 3–5 replicates for each donor, and different donors are represented by different symbols (n = 3). P-values are from post-hoc pairwise comparisons of marginal means based on beta generalized linear models (***p<0.001). **C **Same experiments as (**B**), GFP expression levels in PMA/I stimulated and unstimulated T cells co-cultured with IEC −.

Most CD4 + T cells in the intestinal mucosal area are in an activated state [34]. There is also some evidence that activated CD4 + T cells may also play a role in HIV persistence [45]. Therefore, we sought to investigate the effects of IEC stimulation on the infection of activated CD4 + T cells. PBMC from HIV negative donors were activated using phytohemagglutinin (PHA, 5 µg/mL) and IL-2 (10ng/mL). Two days after activation, total CD4 + T cells were isolated and cultured in IL-2 (10ng/mL) containing media. On day 6 post-activation the CD4 + T cells were cultured alone (ACT) or co-cultured with the IECs (IEC − and IEC + ), and on day 7 post-activation the cells were infected with the GFP reporter virus. Infection rates were examined 3 days post-infection. We found that IEC stimulation dramatically increased HIV infection rates in activated CD4 + T cells (Fig. 5A, p < 0.0001). Infection levels in IEC-stimulated CD4 + T cells can achieve 40–50%, compared with < 10% in unstimulated CD4 + T cells.

### IEC induce latent infection in activated CD4 + T cells

From our previous work, we observed that activated CD4 + T cells do not harbor latent infection after in vitro infection [37]. In this study, we decided to examine whether activated CD4 + T cells would harbor latent infection after IEC stimulation. To do so, we followed the same procedure as with productive infection: PHA-activated CD4 + T cells were cultured alone or co-cultured with IEC on day 6 post-activation, and on day 7 post-activation, they were infected with the GFP reporter virus. We chose to infect activated CD4 + T cells on later days post-activation (on day 7 rather than on day 3 peak activation) because there was a study showing infection on later days post-activation generated more latent infection [19]. On day 5 post-infection, when GFP expression had plateaued, productively infected (GFP +) cells were removed and GFP − cells were collected via flow cytometric sorting. The GFP- cells were then cultured with or without PMA/I treatment. To prevent de novo integration and to ensure post-integration latency was detected, an integrase inhibitor Raltegravir was included in the culture media for both PMA/I treated and untreated CD4 + T cells. Compared with IEC-stimulated CD4 + T cells without PMA/I, PMA/I treatment significantly increased GFP expression in IEC-stimulated T cells (Fig. 5B, p = 0.0003 for both IEC − and IEC + stimulated T cells), demonstrating that latent infection was present in activated CD4 + T cells stimulated by IEC − and IEC +. In contrast, in activated CD4 + T cells cultured alone, PMA/I treatment did not increase GFP expression (p = 0.99), confirming the lack of latent infection (Fig. 5B). Figure 5C shows a flow cytometry plot comparing GFP expression levels in PMA/I stimulated versus unstimulated T cells co-cultured with IEC −.

### IEC increase productive R5-tropic virus infection in resting memory CD4 + T cells and activated CD4 + T cells

**Figure 6:**
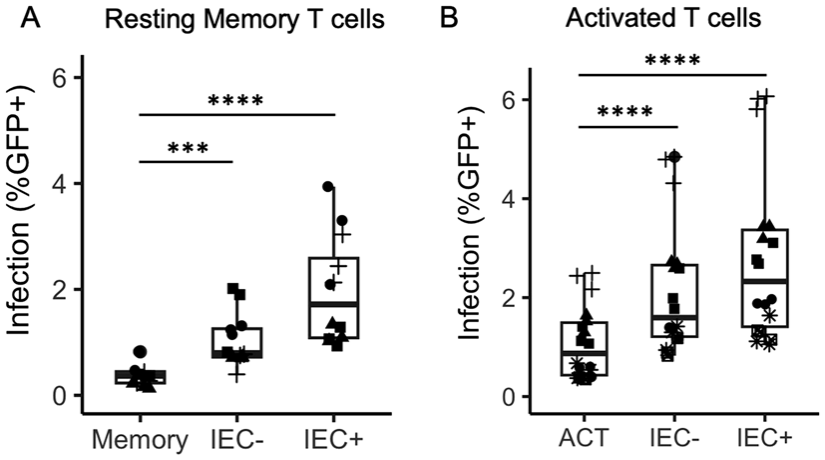
**Fig. 6** IEC increase productive R5 infection in resting memory T cells and Activated T Cells. **A** Infection of R5 tropic virus in resting memory T cells. CD4 + resting memory T cells were isolated from PBMC and cultured alone or co-cultured with IEC for a day, then they were infected with an R5 tropic reporter virus. GFP was measured on day 6 post-infection. **B** Infection of R5 tropic virus in activated CD4 + T cells. PBMC were activated with PHA (1 mg/mL) for 2 days before isolating CD4 + T cells by bead depletion. On day 6 post-activation, the activated CD4 + T cells were then cultured alone, or co-cultured with IEC −, or IEC +. All CD4 + T cells were infected with an R5 tropic HIV reporter virus expressing GFP one day after co-culture. GFP expression was measured on day 3 post-infection. Samples were taken in triplicate for each donor, and different donors are represented by different symbols (**A** n = 4, **B** n = 6). P-values are from post-hoc pairwise comparisons of marginal means based on beta generalized linear models (***p<0.001, ****p<0.0001).

Most CD4 + T cells in the gut mucosal area are of activated and memory phenotype [34]. Both activated and memory CD4 + T cells are known to express more CCR5 [46], so we investigated whether IEC have a similar effect with R5-tropic virus as with X4-tropic virus, which was the virus we had been using so far. The experimental procedures were very similar to those using the X4 tropic virus. We isolated resting memory CD4 + T cells from PBMC and cultured them alone or co-cultured them with IEC for a day, then we infected them with an R5 tropic reporter virus (identical to the X4 virus except for the Env protein). Infection (%GFP +) levels were measured 6 days post-infection. We found that resting memory CD4 + T cells co-cultured with IEC had significantly higher infection rates than resting memory CD4 + T cells cultured alone. (Fig. 6A, p < 0.001 and p < 0.0001 for IEC − and IEC + respectively). We also investigated whether IEC stimulation would increase R5 tropic virus infection of activated CD4 + T cells. PHA-activated CD4 + T cells were cultured alone or co-cultured with IEC on day 6 post-activation, and on day 7 post-activation, they were infected with the R5 tropic GFP reporter virus. Infection rates were examined 3 days post-infection. As shown in Fig. 6B, IEC stimulation significantly increased productive R5 virus infection in activated CD4 + T cells (p < 0.0001 for both IEC − and IEC +).

### IL-6 is not involved in IEC induction of productive infection in activated CD4 + T cells

**Figure 7:**
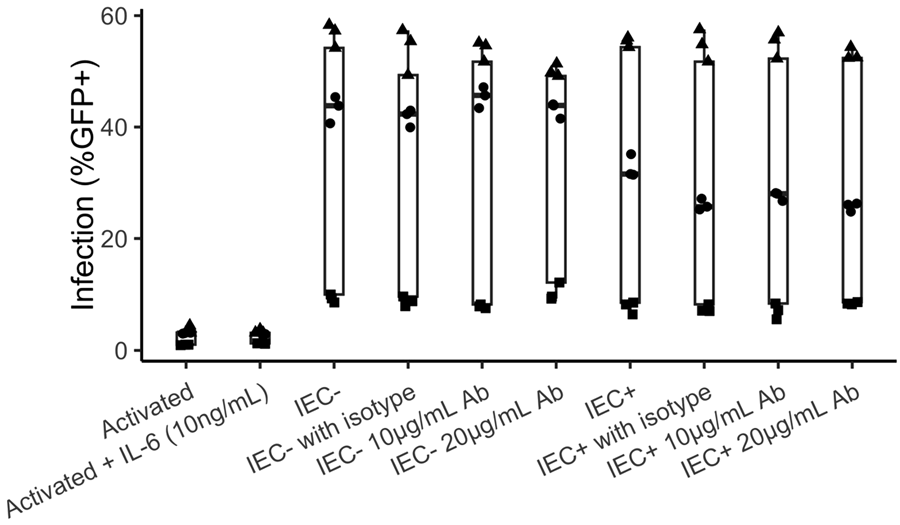
**Fig. 7** IL-6 is not involved in IEC induction of productive infection in activated CD4 + T cells. PBMC were activated with PHA (1mg/mL) and IL-2 (10ng/mL) for 2 days before isolating CD4 + T cells by bead depletion. On day 6 post-activation, the activated CD4 + T cells were either cultured alone, cultured with recombinant IL-6 (10ng/mL), or co-cultured with IEC −, or IEC +, with or without isotype control antibody (20µg/mL) or anti-IL-6 antibody (10 and 20µg/mL). All CD4 + T cells were infected with an HIV reporter virus expressing GFP one day after co-culture. Infection (%GFP +) rates were measured 3 days post-infection. Samples were taken in triplicate for each donor, and different donors are represented by different symbols (n = 3). P-values from post-hoc pairwise comparisons of marginal means based on beta generalized linear models were not significant between isotype controls and IL-6 Ab treatments, or between activated T cells and those treated with IL-6 cytokine.

IL-6 was found to be involved in IEC induction of productive infection in resting CD4 + T cells (Fig. 2A). We sought to test whether IL-6 was involved in IEC stimulation of activated CD4 + T cells as well. Activated CD4 + T cells isolated from PHA-activated PBMC were cultured alone, with 10ng/mL recombinant IL-6, or co-cultured with IEC +/− on day 6 post-activation. In some wells with IEC co-culture, an isotype control antibody (20 µg/mL) or anti-IL-6 antibody (10 and 20 µg/mL) was added at the time of co-culturing. One day later, all wells were infected with the GFP reporter virus, and infection (%GFP +) rates were measured 3 days post-infection. Recombinant IL-6 was replenished one day after infection. As seen in Fig. 7, recombinant IL-6 did not increase infection level in activated CD4 + T cells. Anti-IL-6 antibody also had no effect in reducing infection rates in IEC stimulated activated CD4 + T cells.

### IEC increase productive infection in CCR6 + Th 17 cells

**Figure 8:**
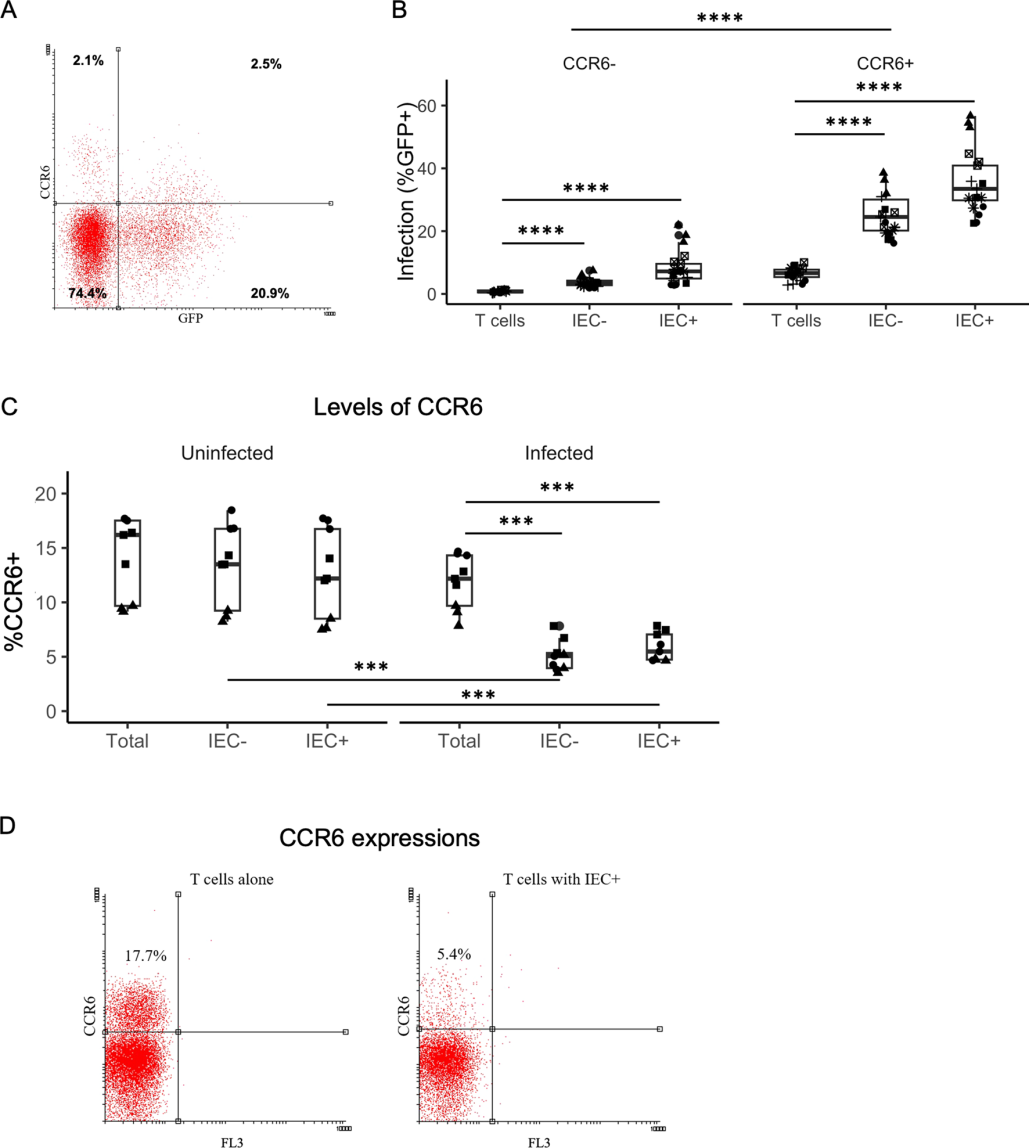
**Fig. 8** Productive Infection in CCR6 + CD4 + T cells co-cultured with IEC. Total CD4 + T cells were isolated from PBMC either cultured alone or with IEC +/− for one day, and then infected with an HIV reporter virus expressing GFP one day after co-culture. Infection (%GFP +) rates were measured 6 days post-infection, along with CCR6 + levels by antibody staining and flow cytometry. **A** FACS plot showing CCR6 staining vs. GFP from an IEC + T co-culture well. **B** Infection (%GFP +) rates in CCR6 + and CCR6 − T cells cultured alone or in IEC +/- co-cultures. **C** The total percentage of CCR6 expressing T cells in each culturing condition: T cells alone, co-cultured with IEC − or IEC +, whether infected or uninfected. **D** FACS plot showing % CCR6 + cells in T cell cultured alone vs. co-cultured with IEC + (both infected). **B** and **C** Samples were taken in triplicate for each donor, and different donors are represented by different symbols (**B** n = 7, **C** n = 3). P-values are from post-hoc pairwise comparisons of marginal means based on beta generalized linear models (***p<0.001, ****p<0.0001).

A significant CD4 + T cell population in the intestinal mucosal area is Th17 cells. During HIV infection, large numbers of Th17 cells are infected and depleted from the gut environment [40, 41]. We sought to investigate the effect of IEC stimulation on HIV infection of Th17 cells. Th17 cells were distinguished from the rest of the CD4 + T cell population by the marker CCR6, a chemokine receptor involved in T cell homing to the intestinal mucosal area [39]. Many Th17 cells in the gut environment exhibit activated phenotypes, while some are resting, so we examined both activated and resting CD4 + T cells isolated from PBMC for the effect of IEC stimulation. Total (both activated and resting) CD4 + T cells were isolated from PBMC and cultured alone or co-cultured with IEC +/− for a day. Half of the wells in each culturing condition were infected, while half the wells were left uninfected. 6 days post-infection, all cells were stained with anti-CCR6-APC antibodies, and flow cytometry was used to assess CCR6 expressions along with the infection (%GFP +) levels (Fig. 8A). CCR6 + T cells showed a higher infection rate than their CCR6- counterparts, whether cultured alone or co-cultured with IEC (Fig. 8B, overall comparisons p < 0.0001). Although IEC stimulation increased HIV infection rates in both CCR6 − and CCR6 + T cells (Fig. 8B, comparisons between IEC stimulated T cells versus T cells alone, p < 0.0001), in CCR6 + T cells there was a greater increase in infection rates than in CCR6- T cells. IEC- stimulation increased infection by > 18% on average in CCR6 + T cells compared with 3% in CCR6-T cells; likewise, IEC + stimulation increased infection by > 29% on average in CCR6 + T cells compared with 7.5% in CCR6 − T cells. The CCR6 + T cells stimulated by IEC + showed the highest levels of infection overall: as high as > 50% (Fig. 8B).

Levels of CCR6 + cells were compared between infected and uninfected total CD4 + T cells on day 6 post infection, whether cultured alone or in IEC co-cultures. There was a slight decrease of CCR6 + cells in T cells cultured alone after HIV infection (not statistically significant), but the decrease was substantially more pronounced in IEC co-cultures (Fig. 8C, p < 0.001). Compared with infected T cells cultured alone, those T cells co-cultured with IEC +/− showed a substantial reduction of CCR6 + cells (Fig. 8C, p < 0.001 and D). Because this drop in CCR6 + cells was not observed in uninfected conditions upon IEC stimulation, we hypothesized that upon IEC stimulation, there was preferential death of CCR6 + cells from HIV infection.

## Discussion

Intestinal mucosal tissue has been a major site of HIV infection, pathology, and persistence [4]. Dramatic loss of CD4 + T helper cells, particularly the Th17 subpopulation, contributes greatly to HIV pathogenesis and the demise of the immune system [40, 41]. The tissue microenvironment in the gut plays an important role in HIV infection and pathology. Endothelial cells are abundant in the intestinal tissue microenvironment. In this study, we investigated the role intestinal endothelial cells (IEC) play in HIV infection of CD4 + T cells.

We found that upon stimulation of IEC, HIV infection in resting CD4 + T cells had increased substantially without showing CD4 + T cell activation (Fig. 1B and C), indicating the mechanism of increased HIV infection in resting CD4 + T cells was not due to cell activation, or at least not to the level that can be measured by activation markers. In this case, CD4 + T cell activation was measured by antibody staining of activation markers CD25, CD69 and HLA-DR, and more sensitive measures such as scRNA-Seq were not used. IEC-induced increase in HIV infection is more prominent in memory CD4 + T cells compared with naïve CD4 + T cells (Fig. 1D). EC are known to stimulate memory CD4 + T cells better than naïve CD4 + T cells in vivo, coinciding with the finding that most latent reservoirs exist in memory CD4 + T cell populations in HIV infection [47–49]. While resting CD4 + T cells form low levels of latent infection, upon IEC stimulation, the levels of latent infection were substantially increased (Fig. 4). Some thought resting CD4 + T cells could not be infected directly, and a latent reservoir must have formed when infected activated CD4 + T cells reverted to a resting state [17–19]. Others have shown that chemokines and cell-cell interactions can promote HIV infection of resting CD4 + T cells, including latent infections [50–53]. We have demonstrated in our EC stimulation model that EC could render resting CD4 + T cells permissible for HIV infection, including latent infection [28, 30]. Here we showed again the importance of endothelial cells, particularly the intestinal context, in latent reservoir formation in resting CD4 + T cells.

Most T cells in the intestinal environment exhibit an activated phenotype, which would make them more prone to experience productive infection rather than forming latent infection. Some have found that productively infected activated CD4 + T cells decay rapidly and are unlikely to form latent reservoir [1, 54], and others showed latent infection occurs in non-dividing CD4 + T cells rather than activated and proliferating CD4 + T cells [35]. However, the GALT has been identified as a major latent reservoir even for patients suppressed on long-term anti-retroviral therapy [13], and latent infection was found in cells isolated from the GALT [55, 56], even though they exhibit activated phenotypes. In our previous study, we activated CD4 + T cells from PBMC and infected them at the peak of activation (3 days post-activation), and we found no evidence of latent infection in these activated CD4 + T cells [28]. A study by Shan et al. showed that infecting activated CD4 + T cells past the peak of activation resulted in more latent infection [19]. In this study, we infected in vitro activated CD4 + T cells at day 7 post-activation rather than day 3, and we still did not detect latent infection (Fig. 5B). However, upon stimulation by IEC, there were significantly increased levels of latent infection (Fig. 5B). This finding suggests a mechanism for HIV to persist and to form a latent reservoir in the activated CD4 + T cells in the gut mucosal area. It could help explain the observations that even though there were more activated CD4 + T cells in the intestinal mucosal area compared to PBMC, there were more latently infected cells [56].

Not only did IEC induce *latent* infection in activated CD4 + T cells, but they also substantially increased the level of productive infection in activated CD4 + T cells (Fig. 5A). Activated CD4 + T cells are known to harbor higher levels of productive infection than resting CD4 + T cells (compare Figs. 1C and 5A for cells cultured alone), but the level of infection can be further increased by IEC stimulation (Figs. 5A and 7), and often quite dramatically. This was true for CD4 + T cells activated via mitogen (PHA) in vitro (Figs. 5A and 7) as well as for activated CD4 + T cells isolated from PBMC (data not shown). Such increase of infection would probably contribute significantly to the infection and massive depletion of CD4 + T cells in the gut mucosal area during primary HIV infection.

T cells in the intestinal mucosal area exhibit activated and memory phenotype, including increased expression of the chemokine receptor CCR5 [46]. CCR5-tropic HIV is the dominant subtype during primary infection [36]. Since the gut mucosal area is a major site of HIV replication during primary infection, we used a CCR5-tropic GFP reporter virus to test infection in activated and memory CD4 + T cells with or without IEC stimulation. Just as was the case for X4-tropic virus (Figs. 1D and 5A), IEC stimulation also increased infection with R5-tropic virus in activated and memory CD4 + T cells (Fig. 6).

As to the mechanism of IEC stimulation of resting CD4 + T cells, we found that IL-6 played a crucial role. IL-6 was necessary for the effect of IEC stimulation as demonstrated by the antibody blocking experiments (Fig. 2A), but it was not sufficient to reconstitute the full effect of endothelial cells [29]. More interestingly, IL-6 did not seem to have any role in IEC’s effect on activated CD4 + T cells (Fig. 7). Recombinant IL-6 cytokine did not increase infection in activated CD4 + T cells by itself, nor did the anti-IL-6 antibody interfere with IEC stimulation. There must be another mechanism in play for activated CD4 + T cells. A recent paper by Card et al. discovered that integrins LFA-1 (αLβ2) and VLA-4 (α4β1) played a role in interactions between endothelial cells and CD4 + T cells [33]. Antibodies against integrins blocked the increase of HIV infection in EC-stimulated resting CD4 + T cells. This could be a potential mechanism to be explored for both resting and activated CD4 + T cells. Another potential mechanism could involve the integrin α4β7 and MadCAM, since IEC express MAdCAM, and MAdCAM costimulation through integrin α4β7 was known to promote HIV infection of T cells [57], particularly in CCR6 + T cells [58, 59].

Physiologically peripheral blood CD4 + T cells bearing the chemokine receptor CCR6 traffic to the gut mucosal area. We specifically investigated CD4 + CCR6 + T cells for the effect of IEC stimulation during HIV infection. We found that CCR6 + T cells were preferentially infected, as was shown [43], but the infection was substantially increased and often to very high levels upon IEC stimulation, particularly with IEC + (Fig. 8B). In addition, there seemed to be a preferential depletion of CCR6 + T cells upon infection, as infected T cells in IEC co-cultures have a much lower percentage of CCR6 + T cells (Fig. 8C). There was no drop of CCR6 + T cells in IEC co-culture without infection, suggesting the drop was not due to IEC stimulation of CD4 + T cells only. We suspect the drop in CCR6 + cells in IEC stimulated infected T cell cultures was due to preferential cell death of CCR6 + cells after infection. If this is true, then it helps explain the in vivo observations: Th17 cells were preferentially depleted during HIV primary infection [40, 41]. One can imagine CD4 + CCR6 + T cells from the peripheral blood travel to the gut mucosal area, encounter IEC in the gut microenvironment, and are now primed for HIV infection. During primary infection, mass numbers of such T cells are infected in the gut, and such infected T cells are more prone to die after IEC stimulation, causing massive depletion from the gut mucosal area.

Using our IEC and CD4 + T cell co-culture system, we have demonstrated the significant effect of IEC on HIV infection of CD4 + T cells. However, such in vitro co-culture systems cannot completely replicate the complex and dynamic interactions between EC and CD4 + T cells in vivo. Further studies in more physiologically relevant settings, such as in ex vivo or in vivo studies, might be needed to confirm the findings and to fully understand the biological processes involved in the interaction between EC and CD4 + T cells.

## Conclusions

In this study, we demonstrated that IEC played a significant role in HIV infection and pathogenesis of the CD4 + T cells. After IEC stimulation, the increase of HIV infection in both resting and activated CD4 + T cells was substantial and sometimes dramatic, particularly in CCR6 + Th17 cells, which are abundant in the gut mucosal area. The level of infection after IEC stimulation was more substantial in memory CD4 + T cells than naïve CD4 + T cells, correlated with the in vivo observation that memory CD4 + T cells harbor much more HIV infection than naïve CD4 + T cells. The mechanism of IEC stimulation in resting CD4 + T cells involved IL-6, but not the co-stimulatory molecule CD2 or any of the six cytokines shown to be elevated in IEC co-culture compared with CD4 + T cells alone. In addition, latent infection was elevated upon IEC stimulation of resting CD4 + T cells, whereas in activated CD4 + T cells, IEC stimulation enabled latent reservoir formation. This is the first time IEC are implicated in HIV infection and pathology in the gut. As the intestinal mucosal area is a major site of HIV pathology and latency formation, this study highlights the importance of lymphoid tissue environment in HIV disease and persistence, particularly the involvement of EC in the gut microenvironment as a significant player.

### Methods

#### Study participants and ethics statement

Healthy blood donors were recruited locally in Grand Rapids Michigan. Both men and women between the ages 18 and 70 were recruited. All participants signed informed consent forms. The study protocol was approved by the Institutional Review Board of Calvin University (#20-023).

#### Endothelial cells, antibodies, and in vitro infection assays

Three different types of endothelial cells were used in this study: human intestinal endothelial cells (IEC), human lymphatic endothelial cells (LEC) and human umbilical vein endothelial cells (HUVEC or EC). IEC (Human Intestinal Microvascular Endothelial Cells—10HU-065) were obtained from iXCells Biotechnologies (San Diego, CA), which were isolated from human intestine specimens, and cultured in basal endothelial cell medium (iXCells) supplemented with Endothelial Cell Growth Supplement (I-ECGS), and 1% penicillin/streptomycin (P/S) (Invitrogen). LEC were obtained from ScienCell Research Laboratories (isolated from human lymph nodes) and cultured in basal endothelial cell medium supplemented with 5% fetal bovine serum (FBS) and 1% penicillin/streptomycin solution (P/S) (Invitrogen). EC were purchased from PromoCell (Germany) and cultured in M199 media supplemented with 20% FBS and 1% P/S. Lymphatic endothelial cell growth factors (ScienCell) were added to LEC, and endothelial cell growth factors (BD Biosciences) were added to EC fresh every 3 days to a final concentration of 50 µg/mL. When indicated as “+”, all four types of endothelial cells were pre-treated with IFN-γ (50 ng/mL) (Invitrogen) for 3 days prior to the addition of resting T cells, which induced the expression of MHC class II. Endothelial cells were plated to 100% confluence and 300,000 resting T cells were co-cultured with IEC/LEC/EC per well of a 24-well plate, or up to 8 million T cells per well in a 6-well plate. Resting T cells were co-cultured with IEC/LEC/EC for 1 day in RPMI + 10% FBS + 1% P/S antibiotics (without IEC/LEC/EC growth factor or IFN-γ) prior to overnight infection. The co-cultures were maintained in the same media for the duration of the experiments. Expressions of GFP and T cell activation markers were examined on various days post-infection using flow cytometry.

In experiments involving activated T cells, PBMC were activated with phytohemagglutinin (PHA, 5 µg/mL, Sigma) and IL-2 (10 ng/mL, or approximately 200U/mL, BioLegend) for 2 days prior to a negative bead depletion to isolate total CD4 + T cells. The activated T cells were co-cultured similarly to the resting T cells in the manner described above with addition of IL-2 (10 ng/mL) to the culture media.

Antibodies for various activation markers (CD25, CD69 and HLA-DR) were purchased from BioLegend and used according to manufacturer’s recommendations. Anti-human CCR6 antibodies -APC were obtained from BD Bioscience and used according to manufacturer’s recommendations.

#### Virus production

NL43-dE-GFP reporter viruses were generated by cotransfecting HEK293T cells with a plasmid encoding NL43-dE-GFP and a plasmid encoding the HIV-1 envelope (pWE-CXCR4 for X4 tropic virus and pSF162 for R5 tropic virus) using TrueFect (United Bio-systems) at a 2:1 ratio (pNL43:pWE/pSF162). All three plasmids were gifts from Dr. Robert Siliciano at Johns Hopkins University. Supernatants were collected after 72 h and filtered through a 0.22 μm membrane to remove cell debris. Virus particles were pelleted using a Lenti-X concentrator (Clontech Laboratories) by following the manufacturer’s instructions and resuspended with 1/27 of the original volume of RPMI + 10% FBS.

#### Separation of various T Cell populations

Human peripheral blood mononuclear cells (PBMC) were obtained from HIV- blood by centrifugation through a Ficoll-Hypaque density gradient at 300 x *g* for 60 min. Activated CD4 + T cells were purified from PBMC using Miltenyi microbeads (a negative depletion kit for isolating CD4 + T cells). Resting CD4 + T cells were purified from PBMC similarly with the addition of biotin-labeled anti-CD25 (Miltenyi or Raybiotech) and anti-HLA-DR antibodies (BioLegend) to the Miltenyi depletion cocktail mix. CD45RO+/CD45RA- memory T cells and CD45RO−/CD45RA+ naïve T cells were also purified using their respective Miltenyi negative depletion kits. Similarly, biotin-labeled anti-CD25 and anti-HLA-DR antibodies were added.

#### Detection of latent infections

For experiments on latent infections, as described previously [28], flow cytometric sorting was done to remove productively infected (GFP +) T cells, at 8 days post-infection for resting T cells, and at 5 days post-infection for activated T cells. After sorting, the GFP negative cells were cultured with or without phorbol myristate acetate (PMA (10ng/mL)) plus Ionomycin (1 µg/mL) (both from Sigma) for 1 day. Integrase inhibitor Raltegravir (3.3mM, Selleck) was added to prevent de novo integration. On the second day post-sorting, flow cytometric analysis of GFP expression was conducted.

#### Blocking IL-6 and CD2

Antibodies were used to block the effects of IL-6 and CD2 signaling in the T cell and IEC co-cultures. When blocking IL-6, LEAF-Purified anti-human IL-6 antibody (BioLegend) was added to the wells at various concentrations immediately after introducing CD4 + T cells to IEC. For CD2 blocking, resting CD4 + T cells were incubated with LEAF-Purified anti-human CD2 antibody (BioLegend) at various concentrations for 1 h prior to being co-cultured with IEC. For both IL-6 and CD2 blocking, the antibody was refreshed 1-day post-infection. Infection levels were measured 6 days after infection for resting T cells and 3 days after infection for activated T cells.

#### T cell stimulation with FGF-2, CXCL1, CXCL10, PDGF-BB, TGF- α, CCL5, and IL-6

Resting T cells were treated with recombinant human FGF-2, CXCL1, CXCL10, PDGF-BB, TGF-α, CCL5 (BioLegend) at 3ng/mL, and/or with recombinant human IL-6 (BioLegend) at 10 ng/mL, which is at least 3 fold higher than the concentrations in EC cultures determined by the multiplex enzyme-linked immunosorbent assay (ELISA) to ensure the potency of recombinant cytokines [29]. After incubation with the cytokines for 1 day, the T cells were infected with the reporter virus. Cytokine levels were refreshed 1, 3, and 5 days after infection, and the cells were examined for infection levels on day 6 post-infection for resting T cells or day 3 for activated T cells.

#### Statistical analyses

To assess differences between cell types and treatments while controlling for donor-to-donor variation, we fitted generalized linear models. All models were fit in R statistical computing software [60] using package glmmTMB [61]. Since infection rates are bounded between 0 and 100%, models used the beta family with a logit link function; each model included fixed effects of cell type or treatment variables as well as donors. Type II ANOVA was used to test for differences between cell types or treatments (using R package car [62]), followed by post-hoc pairwise comparisons of marginal means computed with R package emmeans [63], using Tukey’s method for adjusting p-values for multiple comparisons. Data in Figs. 1B, C, 2B, C, 4C, 5A, B, 6 A, B were analyzed based on all donors and all replicates within each donor. Data in Figs. 1A, D, 2A, 3B, 4B, 7, 8B and C were analyzed by first averaging replicates within each donor to meet model conditions. Remaining figures present data from single donors.

## Data Availability

The datasets used and/or analyzed during the current study are available from the corresponding author on reasonable request.

## Abbreviations

CCL5: CC motif chemokine ligand 5
CCR5: CC motif chemokine receptor 5
CCR6: CC motif chemokine receptor 6
CD2: Cluster of differentiation 2
CD4: Cluster of differentiation 4
CD25: Cluster of differentiation 25
CD58: Cluster of differentiation 58
CD69: Cluster of differentiation 69
CD80/86: Cluster of differentiation 80/86
CXCL1: CXC motif chemokine ligand 1
CXCL10: CXC motif chemokine ligand 10
CXCR4: CXC motif chemokine receptor 4
EC: Endothelial cells (in general), specifically human umbilical vein endothelial cells where indicated
EC+: Human umbilical vein endothelial cells treated with IFN-γ
EC−: Human umbilical vein endothelial cells not treated with IFN-γ
ECGS: Endothelial cell growth supplement
ELISA: Enzyme-linked immunosorbent assay
FACS: Fluorescence-activated cell sorting
FBS: Fetal bovine serum
FGF-2: Fibroblast growth factor 2
GALT: Gut associated lymphatic tissues
GFP: Green fluorescent protein
HIV: Human immunodeficiency virus
HLA-DR: Human leukocyte antigen—antigen D related
HUVEC: Human umbilical vein endothelial cells
IEC: Intestinal endothelial cells
IEC+: Intestinal endothelial cells treated with IFN-γ
IEC−: Intestinal endothelial cells not treated with IFN-γ
IFN-γ: Interferon gamma
IL-2: Interleukin-2
IL-6: Interleukin-6
LEC: Lymphatic endothelial cells
LEC+: Lymphatic endothelial cells treated with IFN-γ
LEC−: Lymphatic endothelial cells not treated with IFN-γ
MAdCAM: Mucosal vascular addressin cell adhesion molecule
MHC II: Major histocompatibility complex class II
PBMC: Peripheral blood mononuclear cells
PDGF-BB: Platelet-derived growth factor BB
PHA: Phytohemagglutinin
PMA: Phorbol myristate acetate
PMA/I: Phorbol myristate acetate/Ionomycin
P/S: Penicillin/Streptomycin
R5: CCR5 tropic HIV virus
RPMI: Rosewell park memorial institute medium
scRNA-Seq: Single cell RNA sequencing
TCR: T-cell receptor
TGF- α: Transforming growth factor alpha
X4: CXCR4 tropic HIV virus
